# Ecological Preferences and Indication Potential of Freshwater Bryophytes–Insights from Croatian Watercourses

**DOI:** 10.3390/plants11243451

**Published:** 2022-12-09

**Authors:** Anja Rimac, Antun Alegro, Vedran Šegota, Nina Vuković, Nikola Koletić

**Affiliations:** Division of Botany, Department of Biology, Faculty of Science, University of Zagreb, Marulićev trg 20/II, 10000 Zagreb, Croatia

**Keywords:** aquatic bryophytes, autecology, ecological responses, water quality, land use, bioindicators

## Abstract

A comprehensive survey of Croatian watercourses covering the whole of the national territory and investigating inherent watercourse heterogeneity was conducted to explore the ecological responses of the most frequent freshwater bryophytes with respect to water chemistry variables and land use within the catchment area. Direct multivariate ordination (CCA) of vegetation data paired with 18 environmental variables revealed that freshwater bryophytes and their assemblages were segregated along the gradients of water chemistry and the proportion of natural and urban area within the catchment. Generalized additive models (GAM) were employed to explore the ecological responses of individual species. The results showed that most of the investigated species preferred natural, clean, well-oxygenated watercourses, with low nutrient and organic matter content, as well as with low electrical conductivity. Species such as *Palustriella falcata*, *Eucladium vertcillatum*, *Dichodontium flavescens* and *Jungermannia atrovirens* had narrow ecological niches and were restricted to pristine watercourses, while the most frequent and widely distributed species, such as *Fontinalis antipyretica*, *Rhynchostegium riparioides*, *Cratoneuron filicinum*, *Fissidens crassipes*, *Cinclidotus fontinaloides* and *C. riparius*, had a wide ecological tolerance. *Riccia fluitans* and *Leptodyctium riparium* had wide ecological ranges, but with optima in hypereutrophic waters with high nutrient and organic content, as well as high electrical conductivity. Furthermore, these two species were frequently associated with a high share of intensive agriculture and a low share of natural land within the catchment.

## 1. Introduction

Freshwater bryophytes are a common but unassuming and frequently overlooked component of freshwater ecosystems. They constitute an important part of macrophyte vegetation, especially in headwater streams, mountain and upland watercourses, and highly seasonal and intermittent rivers [1,2,3,4,5], and they are the most prominent part of the vegetation of waterfalls and cascades [6,7,8,9]. Bryophytes dominate the vegetation of such lotic habitats due to a wide variety of structural and physiological adaptations [6,10,11]. These make them resilient to seasonal desiccation, high water velocity and associated mechanical stress, which other macrophyte representatives cannot withstand. Freshwater bryophytes play a significant role in these harsh environments as dominant primary producers and influence the overall nutrient and trophic dynamics. Bryophyte beds make an excellent shelter and habitat for various invertebrate assemblages and provide a surface for the growth of epiphytic algae [12,13]. Freshwater bryophytes occur in middle and lower river reaches as well, but in much lower abundance, with the exception of some of the Mediterranean rivers. In middle and lower reaches, they are in general represented by a lower number of rheophyte taxa restricted to larger and thus more stable substrates, or by a somewhat more diverse set of semi-aquatic species inhabiting periodically flooded river margins [14,15].

While the presence and coverage of freshwater bryophytes are primarily determined by riverbed stability and substrate size [16,17,18], physiographic, geological, climatic and water chemistry factors have been identified as major environmental drivers influencing species diversity and composition [1,2,3,16,17,18,19,20,21,22]. Furthermore, changes in stream and river hydrology and morphology, as well as anthropogenically influenced changes in water chemistry, strongly impact individual bryophyte species and whole communities [20,23,24]. Aquatic plant communities, including bryophyte assemblages, have been recognized as valuable indicators of stream biointegrity and water quality [21,25,26,27,28,29,30,31,32] and thus useful in the bioassessment of rivers and lakes. Freshwater habitats have been subject to various anthropogenic pressures in the past decades, with eutrophication and hydromorphological degradation being the most prominent stressors, often acting simultaneously [33,34,35]. They are most often related to urbanization, industrialization, as well as intensive agriculture. Continued stress on the freshwater habitats and its detrimental impact on freshwater biota has resulted, however, in raised awareness of these issues, leading to encouraging progress in the understanding of the ecology of freshwater biota and their communities. This was followed by the development of corresponding biomonitoring methods and legislation frameworks such as the Water Framework Directive (WFD) [28,29,36].

The use of bryophytes as indicators of water quality and ecological status strongly depends on the knowledge of their distribution and ecological responses since the sensitivity of individual species (i.e., the presence or absence of species with narrow ecological niches) or their tolerance (widely distributed with broad niches) to stressors constitutes the basis of biomonitoring [3]. Several studies conducted in Europe have investigated how various physicochemical parameters influence freshwater bryophyte assemblages and how individual species behave along these gradients. Water temperature, pH, conductivity, dissolved oxygen, biochemical oxygen demand and nutrient concentration were identified as the most important among studied water physicochemical parameters [2,16,21,27,30,37,38,39,40,41,42]. Combined stressors, such as intensive agriculture, were recognized as important parameters in the segregation of bryophyte species as well [20]. Furthermore, species richness and total bryophyte abundance were proven to be good metrics indicative of eutrophication and trophic status in general [20,43].

Several systems developed for river monitoring based on aquatic plants include bryophytes as well; e.g., systems developed to monitor trophic status such as the Mean Trophic Rank (MTR) [44,45] and River Macrophyte Nutrient Index (RMNI) [46] developed in the UK, the L’Indice Biologique Macrophytique en Rivière (IMBR) developed in France [47], the Macrophyte Index for Rivers (MIR) originally developed in Poland [48,49], and the Index of Trophy for European Macrophytes (ITEM), which is a pan-European common metric for assessing nutrient enrichment, synthesizing various national macrophyte scoring systems [50]. These systems are based on a list of indicator taxa, while in the assessment of trophic status, average scores of indicative species are weighted by their abundance, with some of the indices taking into account the taxon’s ecological amplitude as well [51]. The majority of included bryophytes are recognized as good bioindicators with narrow ecological tolerance, preferring oligo- and mesotrophic conditions. The abovementioned indices were adjusted and calibrated to meet the requirements of assessing the ecological status of water bodies as required by the WFD. Bryophytes are also listed as indicator species in the German Reference Index (RI) [52,53], which was designed to meet the requirements of the WFD and to indicate non-specific anthropogenic disturbance, not solely trophic status. The basis of this index is the river type-specific definition of reference and non-specific disturbance-indicating taxa. The WFD adopts a more holistic approach to ecological assessment, being based on the structure and function of different biological quality elements in different types of waterbodies, which is philosophically different from traditional approaches to biomonitoring in Europe, and closer to concepts of biotic integrity or ecosystem health [46]. In this context, macrophytes, including bryophytes, have been considered particularly suitable because they are non-mobile and can thus more precisely indicate local changes in the environment, and additionally, they integrate changes over longer periods and successive disturbances, which is especially true for perennials [25,26].

Available research which has quantitatively investigated the relationship of freshwater bryophytes and water physicochemical variables mostly included limited geographic areas and river basins or was concentrated on a particular watercourse type, such as highly seasonal rivers [20]. Additionally, several studies have identified species which may have developed different ecotypes, displaying different ecological behaviour in different river basins and geographic areas [37,38]. For these reasons, further research into the ecology and bioindication potential of this group covering new geographic areas and wide ecological gradients is needed. Prior to our study, no similar work had been conducted in Croatia, and only a few papers on this topic exist from Southeastern Europe, all of them from Bulgaria [14,20,54].

Having this in mind, we conducted comprehensive field research covering the whole of Croatian territory and investigated the inherent watercourse heterogeneity including the karstic watercourses and their characteristic species. Our aims were to:

1. Determine how water physicochemical factors and land use influence species occurrences and segregation;
2. Explore ecological responses of freshwater bryophyte species and augment data on the autecology and ecological preferences of freshwater bryophytes;
3. Infer the bioindication potential of selected species from their optima and ecological tolerance.

## 2. Results

**Figure 1:**
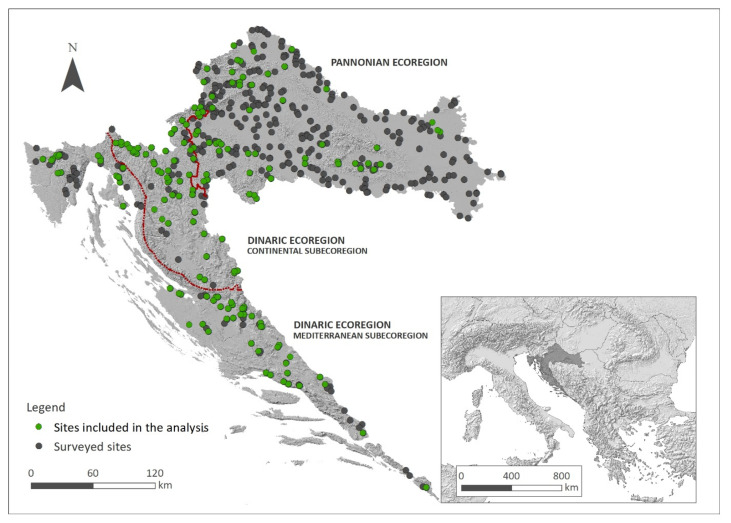
Study area with 648 sampling sites and the 182 sites included in the analysis covering the whole Croatian territory (Southeastern Europe).

A total of 21 freshwater bryophyte species (8 rheophytes, 1 hydrophyte, 2 amphyphytes and 10 hygrophytes) were collected from at least five localities, out of 648 localities surveyed, and were thus included in further analysis. Finally, 182 localities were retained by this criterion, 124 within the Dinaric and 58 within the Pannonian Ecoregion of Croatia (Figure 1). This encompassed diverse watercourse types, from oligotrophic small streams to eutrophic large rivers and artificial canals (Table A1, Appendix A), therefore covering wide gradients suitable for the study of the autecology of selected species. In the Pannonian Ecoregion, the highest bryophyte richness was recorded in small lowland watercourses (16 species). In the Dinaric-Continental Subecoregion, this was true for montane and mid-altitude medium and large watercourses (20 species), followed by montane and mid-altitude small watercourses (18 species). In the Dinaric-Mediterranean Subecoregion, the highest bryophyte richness was recorded in lowland and mid-altitude small watercourses (16 species) (Table A2, Appendix A). As for lowland medium and large watercourses, they harboured a similar set of a total of 11 species and similar overall bryophyte frequency both in the Pannonian Ecoregion and the Dinaric-Mediterranean Subecoregion, while these were substantially higher in the Dinaric-Continental Subecoregion. Species number, as well as their overall frequencies and abundance, in both Dinaric subecoregions exceeded those in the Pannonian Ecoregion (Table A2, Appendix A, Table S1).

Among the most frequent species in our study were *Fontinalis antipyretica*, *Rhynchostegium riparioides*, *Cratoneuron filicnum* and *Leptodyctium riparium*, with the latter being more frequent in the Pannonian Ecoregion, i.e., its lowland watercourses. Species such as *Didymodon tophaceus*, *Eucladium verticillatum* and *Jungermannia atrovirens* were exclusive to the Dinaric Ecoregion, while *Cinclidotus aquaticus*, *Apopellia endiviifola*, *Brachythecium rivulare*, *Dichodontium flavescens* and *D. pellucidum* were more frequent in the Dinaric Ecoregion and within the Pannonian Ecoregion, restricted to only lowland small watercourses.

**Figure 2:**
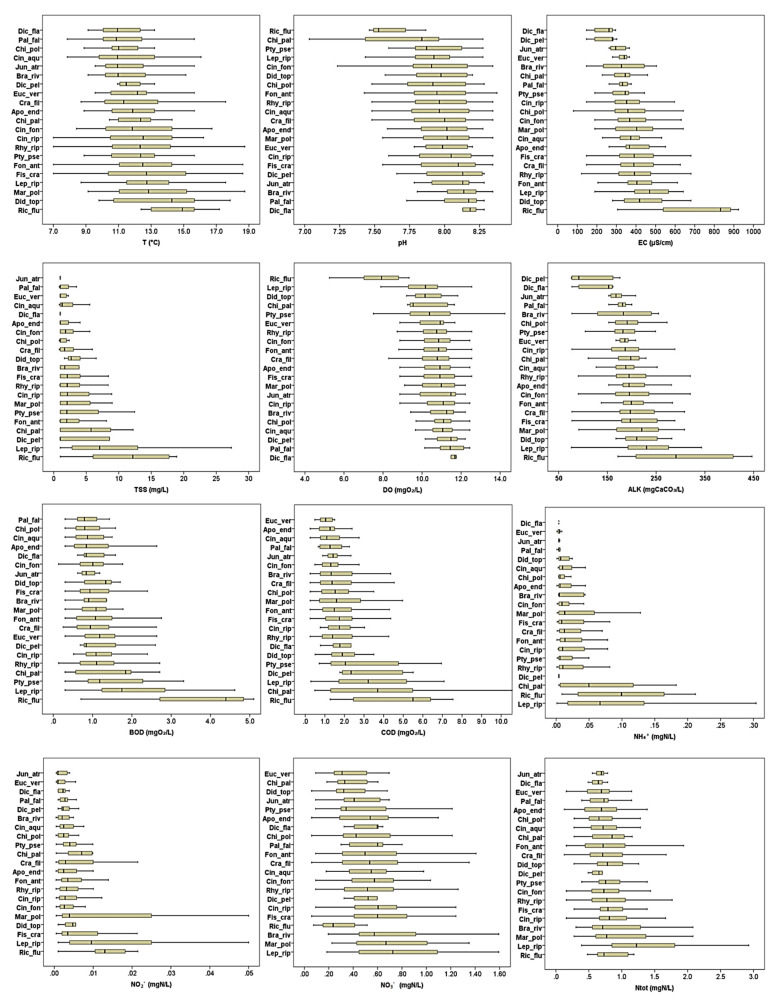

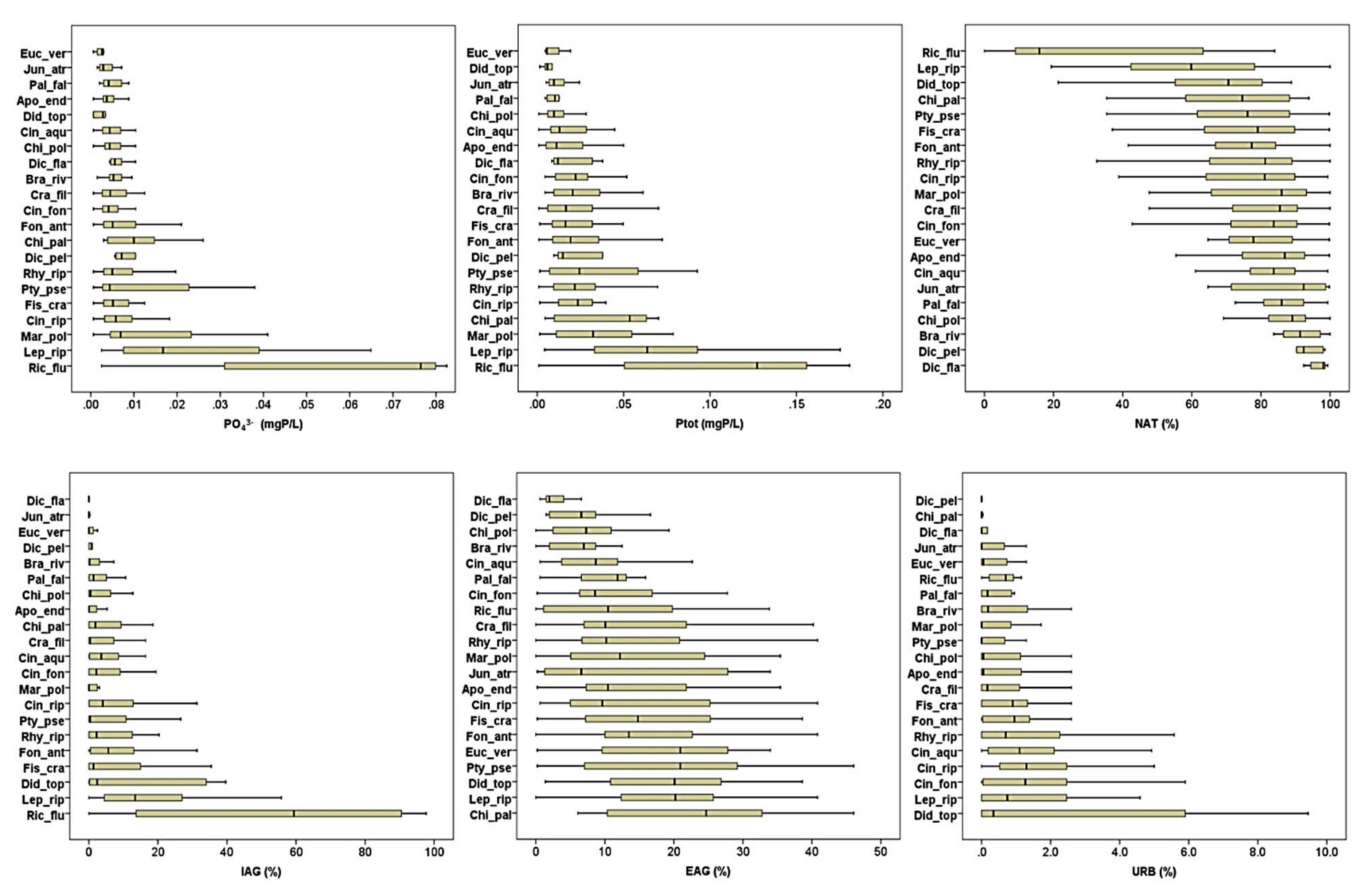
Box-plot graphs for ecological variables within the study for the freshwater bryophytes. For abbreviations of ecological variables see Table 1. Apo end–*Apopellia endiviifolia* (Dicks.) Nebel & D.Quandt, Bra riv–*Brachythecium rivulare* Schimp., Chi pal–*Chiloscyphus pallescens* (Ehrh. ex Hoffm.) Dumort., Chi pol–*Chiloscyphus polyanthos* (L.) Corda, Cin aqu–*Cinclidotus aquaticus* (Hedw.) Bruch et Schimp., Cin fon–*Cinclidotus fontinaloides* (Hedw.) P. Beauv., Cin rip–*Cinclidotus riparius* (Host ex Brid.) Arn., Cra fil–*Cratoneuron filicinum* (Hedw.) Spruce, Dic fla–*Dichodontium flavescens* (Dicks.) Lindb., Dic pel–*Dichodontium pellucidum* (Hedw.) Schimp., Did top–*Didymodon tophaceus* (Brid.) Lisa, Euc ver–*Eucladium verticillatum* (With.) Bruch et Schimp., Fis cra–*Fissidens crassipes* Wilson ex Bruch & Schimp., Fon ant–*Fontinalis antipyretica* Hedw., Jun atr–*Jungermannia atrovirens* Dumort., Lep rip–*Leptodictyum riparium* (Hedw.) Warnst., Mar pol–*Marchantia polymorpha* L., Pal fal–*Palustriella falcata* (Brid.) Hedenäs, Pty pse–*Ptychostomum pseudotriquetrum* (Hedw.) J.R.Spence & H.P.Ramsay ex Holyoak & N.Pedersen, Rhy rip–*Rhynchostegium riparioides* (Hedw.) Cardot, Ric flu–*Riccia fluitans* L.

Species medians revealed the differences in species preferences across the gradients of selected variables, with *Riccia fluitans* and *Leptodyctium riparium* being most obviously separated from the rest of the species in terms of water chemistry and land use variables. These species showed preferences for hypereutrophic water with high nutrient loads, biochemical (BOD) and chemical (COD) oxygen demand and high electrical conductivity, and were more frequently associated with a higher share of intensive agriculture and a low share of natural land within the catchment area (Figure 2). Most other species had their median values in clean water, with lower nutrient content, low BOD, COD and electrical conductivity, indicative of good water quality status. 

### 2.1. Ordination Results

Results of the CCA revealed similar patterns in the ecological preferences of the studied species, except for *Riccia fluitans*, which, as an outlier, was excluded from this analysis. The first axis of the CCA explained 51.06% and the second 16.01% of the variation in the relationship between vegetation data and environmental factors, while eigenvalues of the first and the second axis equalled 0.4 and 0.1, respectively. Forward selection of environmental variables revealed a set of eight most-contributing and non-redundant variables in determining the freshwater bryophyte distribution (Appendix B). Total nitrogen concentration made the largest contribution to explaining the observed variation in bryophyte composition, followed by the share of the natural area within the catchment and chemical oxygen demand. Overall analysis was statistically significant (*p* < 0.002), which was confirmed by the Monte Carlo test (999 permutations). 

**Figure 3:**
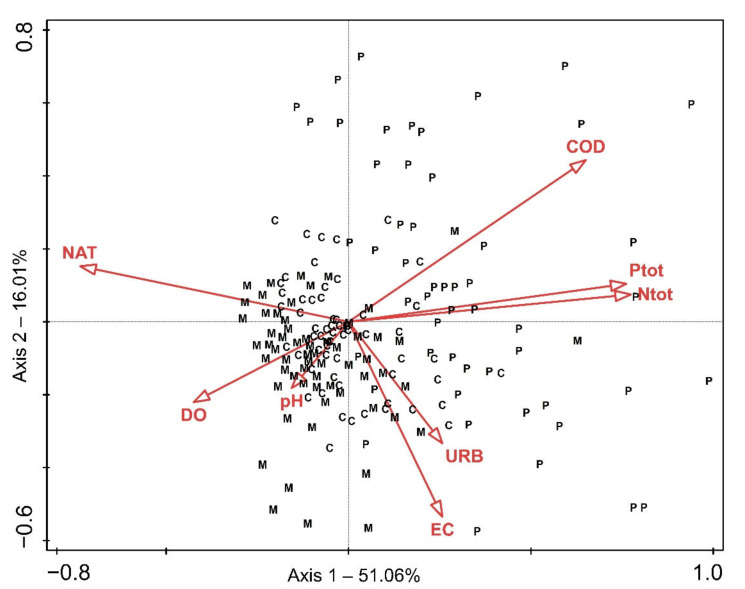
CCA biplot for samples and environmental variables. C—Dinaric-Continental Subecoregion, M—Dinaric-Mediterranean Subecoregion, P—Pannonian Ecoregion, COD—chemical oxygen demand, DO—dissolved oxygen, EC—electrical conductivity, NAT—natural area within the catchment, N_tot_—concentration of total nitrogen, URB—urban area within the catchment.

**Figure 4:**
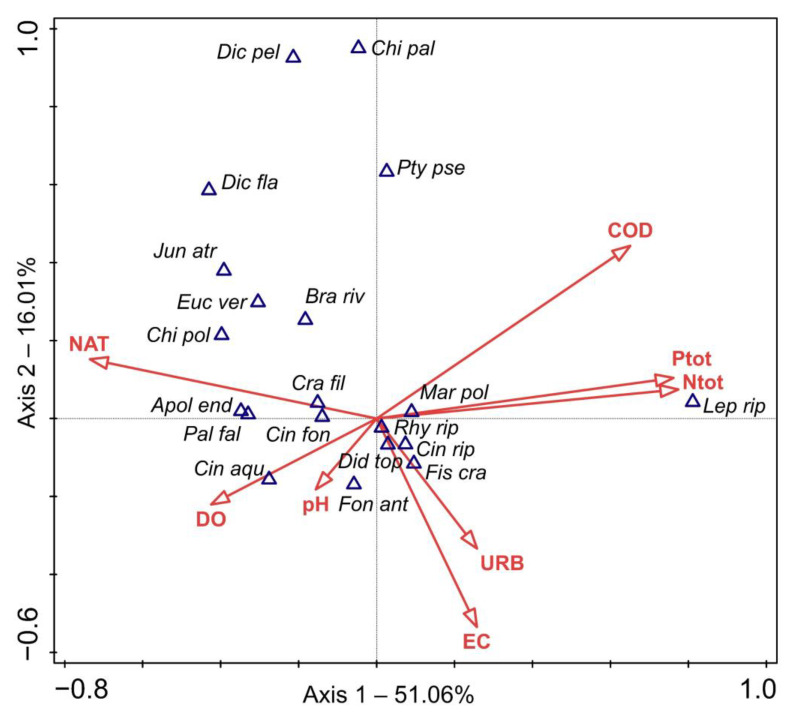
CCA biplot for species and environmental variables. COD—chemical oxygen demand, DO—dissolved oxygen, EC—electrical conductivity, NAT—natural area within the catchment, Ntot—concentration of total nitrogen, URB—urban area within the catchment. For abbreviations of species names, see Figure 2.

The CCA analysis revealed the main compositional gradient representing the water quality along axis 1—from sites with well-oxygenated water, situated within unchanged catchment areas under little anthropogenic influence to sites with high nutrient loads, i.e., high concentration of total nitrogen and total phosphorous, as well as high chemical oxygen demand (Figure 3 and Figure 4). Additionally, the share of the natural area within the catchment was strongly negatively correlated with agricultural land—extensive (AGE, r_s_ = −0.72, *p* < 0.001) and intensive agriculture (AGI, r_s_ = −0.81, *p* < 0.001). Regarding the nutrients, total phosphorus was highly positively correlated with orthophosphates (PO_4_^3−^, r_s_ = 0.82, *p* < 0.001), total suspended solids (TSS, r_s_ = 0.73, *p* < 0.001) and biochemical oxygen demand (BOD, r_s_ = 0.73, *p* < 0.001), and total nitrogen with nitrates (NO_3_^−^, r_s_ = 0.85, *p* < 0.001), while COD correlated with biochemical oxygen demand (BOD, r_s_ = 0.76, *p* < 0.001). Sites with higher nutrient loads, as well as COD and higher shares of agricultural land, were mostly those on the watercourses situated in the Pannonian Ecoregion and were quite well separated from the sites situated in the Dinaric Ecoregion on the CCA ordination biplot, especially those of its Mediterranean Subecoregion (Figure 3). The Dinaric Ecoregion included sites with intermediate values of the studied parameters, as well as sites within natural catchments, with oligotrophic, well-oxygenated water and somewhat higher pH. 

The CCA analysis demonstrated the affinity of particular freshwater bryophyte species to different water chemistry and land use (Figure 4). Mosses such as *Cinclidotus aquaticus*, *Eucladium verticllatum* and *Palustriella falcata*, as well as the liverworts *Apopellia endiviifolia*, *Chiloscyphus polyanthos* and *Jungermannia atrovirens*, preferred clean, well-oxygenated water and were more strongly correlated with a higher share of natural area within the catchment. On the other hand, *Leptodyctium riparium* was of all species investigated the most prominently associated with eutrophic water, while *Cratoneuron filicinum*, *Cinclidotus fontinaloides*, *C. riparius*, *Fontinalis antipyretica*, *Fissidens crassipes* and *Rhynchostegium riparioides* displayed intermediate behavior along the first axis. Among these, *Fissidens crassipes*, *Cinclidotus riparius* and *Rhynchostegium riparioides* were associated with a higher share of urban area within the catchment and higher electrical conductivity.

### 2.2. Species Responses to Environmental Variables

**Figure 5:**
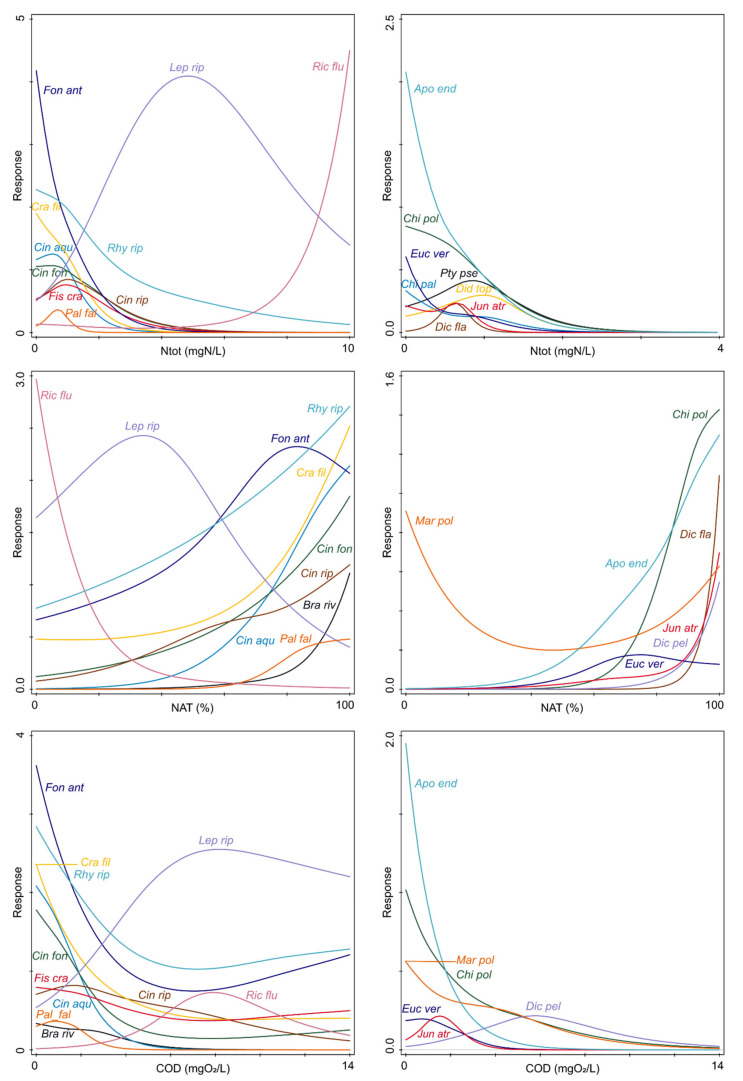

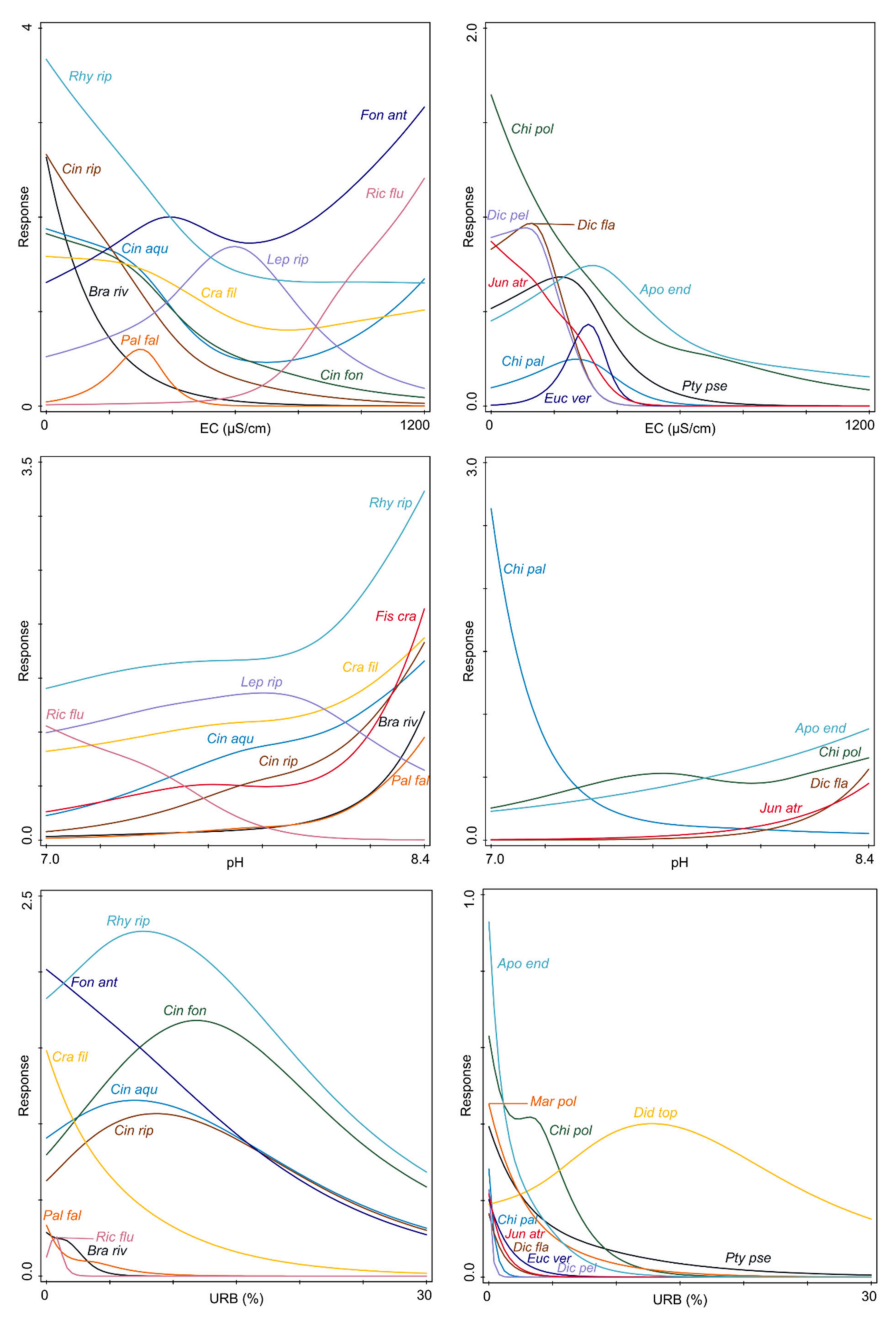

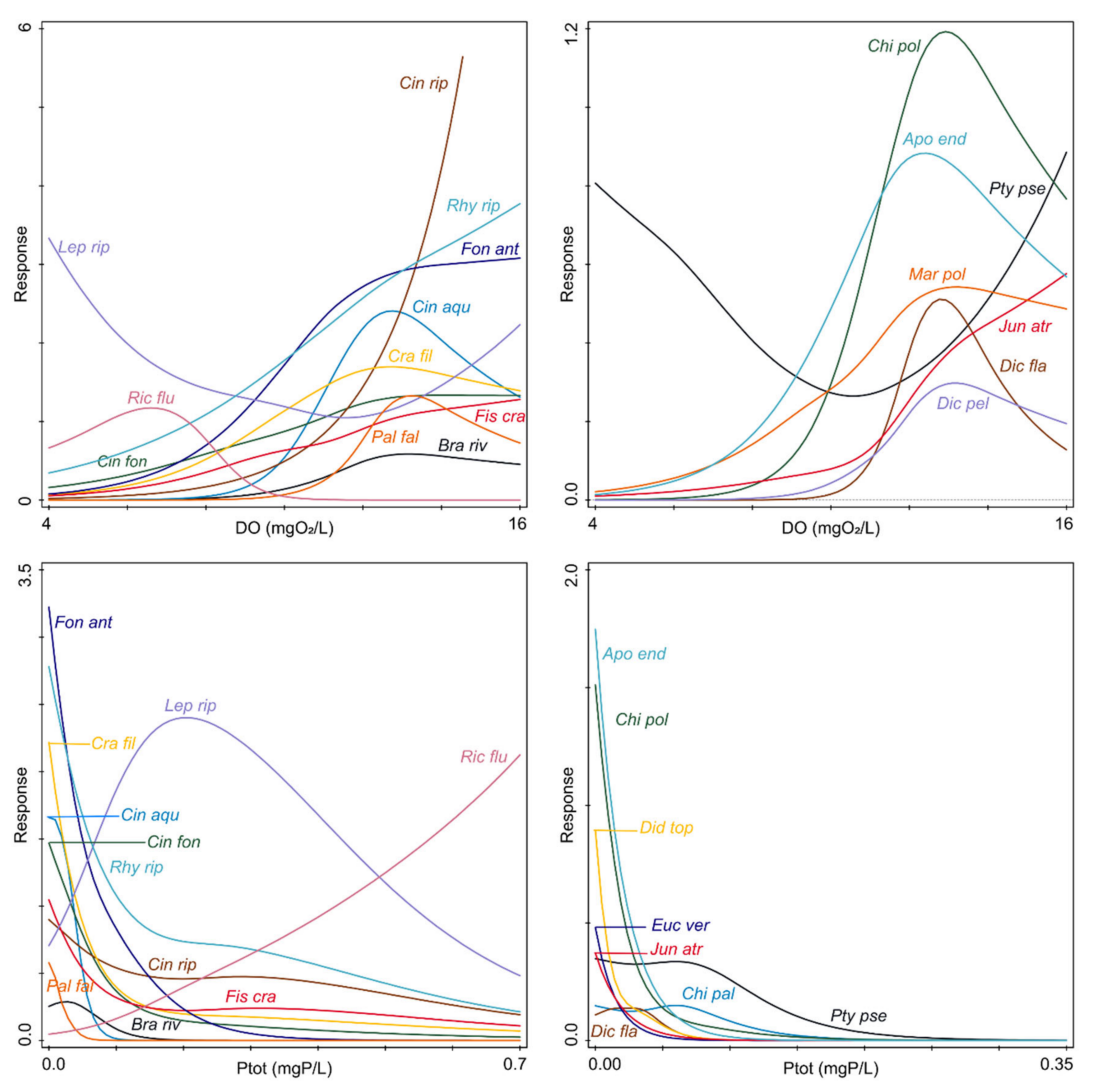
Species response curves for selected environmental gradients (most-contributing according to CCA forward selection) modelled by general additive model. For clarity, response curves of rheophytes, hydrophytes and amphyphytes are shown in graphs on the left and those of hygrophytes on the right side of the figure. Response—abundance estimated on van der Maarel ordinal scale. For species abbreviations, see Figure 2.

The GAM response curves of species abundances for the most influential environmental variables from the CCA additionally corroborated observed patterns (Figure 5). Response curves of GAMs fitted for *Riccia fluitans* and *Leptodyctium riparium* against total nitrogen and total phosphorus showed the preference of both species for hypereutrophic water with a high nutrient load. Regarding total nitrogen, the means of both species were quite high, 1.93 mgN/L for *Riccia fluitans* and 1.45 mgN/L for *Leptodyctium riparium* (Appendix C)*. Leptodyctium riparium* displayed unimodal response with an optimum at 4.90 mgN/L, while *Riccia fluitans* had a monotonically increasing response curve and a maximum at 8.9 mgN/L. Other species had their optima at low levels of total nitrogen (<0.5 mgN/L). A steep decline in abundance with increasing concentrations of total nitrogen was characteristic of most of these species (Figure 5). Species such as *Palustriella falcata*, *Jungermannia atrovirens*, *Dichodotium flavescens*, *Eucladium vericillatum*, *Didymodon tophaceus*, *Chilosyphus polyanthos* and *Apopellia endiviifolia* had quite low maxima and narrow niches, while the maxima of *Cratoneuron filicinum*, *Brachythecium rivulare*, *Cinclidotus fontinaloides*, *C. riparius*, *Fissidens crassipes*, *Fontinalis antipyretica* and *Marchantia polymorpha* were between 2 and 3 mgN/L, and the maximum of *Rhynchostegium riparioides* reached 5.18 mgN/L. These sets of species showed similar behavior with respect to nitrate and ammonium concentration, as well as other water chemistry parameters associated with water quality–orthophosphates, total phosphorus, the amount of organic compounds in water (COD, BOD) and electrical conductivity (Figure 1 and Figure 5).

Regarding the total phosphorus concentration, most of the studied species displayed mean values below 0.04 mgP/L, while the mean values of *Riccia fluitans* and *Leptodyctium riparium* were 0.177 and 0.094 mgP/L, respectively, which corresponded to eutrophic water. The optima of both species were in hypereutrophic water; the optimum of *Leptodyctium riparium* amounted to 0.21 mgP/L, and of *Riccia fluitans* to 0.699 mgP/L (Figure 5). All other species had their optima in oligotrophic water with respect to phosphorus, with *Palustriella falcata*, *Eucladium verticillatum*, *Jungermannia atrovirens*, *Apopellia endiviifolia*, *Chiloscyphus polyanthos*, *Didymodon tophaceus* and *Dichodontium flavescens* having steep monotonically decreasing response curves and low maxima, and *Cinclidotus aquaticus* and *Brachythecum rivulare* tolerating somewhat higher concentrations and disappearing at 0.1 and 0.165 mgP/L, respectively. The abundance of rheophytes such as *Fontinalis antipyretica*, *Cinclidotus fontinaloides*, *C. riparius*, *Fissidens crassipes* and *Rhynchostegium riparioides*, as well as of the amphyphyte *Cratoneuron filicinum* and the hygrophytes *Chiloscyphus pallescens* and *Ptychostomum pseudotriquetrum*, decreased with increasing concentration of total phosphorus, but these species displayed tolerance to eutrophic water. Total phosphorus concentration was highly correlated with total suspended solids, and species preferring, or displaying a high degree of tolerance to, eutrophic water were tolerant of more turbid water as well. These were *Leptodyctium riparium, Fontinalis antipyretica, Rhynchostegium riparioides* and *Riccia fluitans*, persisting at TSS values over 40 mg/L. Nevertheless, the TSS means of all species were below 15 mg/L (Appendix C), i.e., in clear water.

Species response curves for chemical oxygen demand showed that the majority of the species had low optima (<1 mgO_2_/L), while *Leptodyctium riparium* and *Riccia fluitans* peaked at high levels, 8.2 and 7.9 mgO_2_/L, respectively, and *Dichodontium pellucidum* at 4.9 mgO_2_/L (Figure 5). Again, the majority of rheophytes, as well as the amphiphyte *Cratoneuron filicinum*, showed tolerance to high levels of COD, although their abundance decreased with increasing COD. This was also the case with the hygrophyte *Chiloscyphus pallescens*, which was recorded at a maximum COD level of 10.67 mgO_2_/L. GAMs were not successfully fitted for *Didymodon tophaceus* and *Ptychostomum pseudotriquetrum*, but these species tolerated very high COD levels as well, with maxima around 10.6 mgO_2_/L. Species restricted to low levels were *Eucladium verticillatum*, *Jungermannia atrovirens*, *Apopellia endiviifolia*, *Cinclidotus aquaticus*, *Brachythecium rivulare* and *Chiloscyphus polyanthos*. Similar patterns in species preferences can be observed from the descriptive statistics regarding biochemical oxygen demand as well (Appendix C). 

Response curves fitted against dissolved oxygen revealed that the majority of species investigated had their optima in well-oxygenated water (Figure 5). Mean values of this parameter ranged from 10.13 to 11.58 mgO_2_/L, except for *Riccia fluitans.* This species peaked at 6.5 mgO_2_/L, but was present within a quite wide range, from 5.25 to 9.34 mgO_2_/L. The optimum of *Leptodyctium riparium* was at 4 mgO_2_/L, with a prominent decrease in abundance with increasing oxygen concentration. This coincided with the rise in the abundance of other rheophyte species. Nevertheless, *Leptodyctium riparium* was still present in well-oxygenated watercourses, displaying wide tolerance. The set of species restricted to high dissolved oxygen concentration was similar to that characteristic of low nutrient and COD situations. These were *Brachytehcium rivulare*, *Palustriella falcata, Jungermannia atrovirens*, *Dichodontium flavescens*, *Chiloscyphus polyanthos*, *Apopellia endiviifolia* and *Cinclidotus aquaticus*, as well as *Dichodontium pellucidum* and *Chiloscyphus pallescens*. Other species, such as *Rhynchostegium riparioides*, *Fontinalis antipyretica*, *Fissidens crassipes*, *Cratoneuron filicinum*, *Cinclidotus fontinaloides*, *C. riparius* and *Ptychostomum pseudotriquetrum* had wider ranges and were present in less oxygenated watercourses, although in lower abundance. The response curve was not successfully fitted for *Didymodon tophaceus*, but the mean value was at 10.13 mgO_2_/L and the minimum as low as 7.51 mgO_2_/L (Appendix C).

Regarding electrical conductivity, most of the species had optima below 400 μS/cm but displayed different tolerances to higher values. Hygrophyte species had quite narrow niches, with *Apopellia endiviifolia* and *Chiloscyphus polyanthos* and *Ptychostomum pseudotriquetrum* being more tolerant to elevated electrical conductivity than other hygrophytes. Response curves were not fitted for *Dydimodon tophaceus* and *Marchantia polymorpha* but descriptive statistics indicated wide tolerance with respect to electrical conductivity (Appendix C). Similar behavior can be seen in the case of the majority of rheophyte species, except for *Brachythecium rivulare* and *Palustriella falcata*, which were restricted to waters with lower conductivity, while *Rhynchostegium riparioides*, *Cratoneuron filicinum*, *Cinclidotus aqauticus*, *C. riparius* and *C. fontinaloides* showed tolerance to high values of electrical conductivity. The optimum of *Leptodyctium riparium* was around 600 μS/cm, while the abundance of *Fontinalis antipyretica* and *Riccia fluitans* displayed a continuous rise until the maxima above 900 μS/cm (Figure 5). Concerning water pH values, most of the species preferred basic water and showed an increase in abundance above pH 7.9. *Leptodyctium riparium* peaked at 7.87, which was followed by a relatively steep decrease in abundance, which coincided with the increase in the abundance of basophilous species (Figure 5). Furthermore, the abundance of *Riccia fluitans* decreased along the pH gradient and the species disappeared when pH exceeded 7.87. Similarly, *Chiloscyphus pallescens* showed a sharp decrease along this gradient, being most abundant around pH 7, but still present at 8.2. 

GAM response curves fitted against the share of the natural land within the catchment area revealed that the majority of the freshwater bryophytes studied had their optima at values over 80%. Exceptions were again *Riccia fluitans* and *Leptodyctium riparium*. *Riccia fluitans* displayed a steep monotonically decreasing curve with an optimum in the least natural catchments (Figure 5). The optimum of *Leptodyctium riparium* was at 33.57% of the natural area within the catchment but it was present in more natural catchments as well, although in lower abundance. Mean values of this parameter were over 80% for the set of species that were restricted to waters with low nutrient content, low COD, BOD and electrical conductivity, and high dissolved oxygen (*Eucladium verticillatum*, *Apopellia endiviifolia*, *Cinclidotus aquaticus*, *Jungermannia atrovirens*, *Palustriella falcata*, *Chiloscyphus polyanthos*, *Dichodontium flavescens* and *D. pellucidum*), and ranged between 60 and 80% for all other species with wider niches, except for *Riccia fluitans* with a mean value equal to 34.91% (Appendix C). This species was highly associated with intensive agriculture, with the mean value of this parameter being 52.20% and a maximum of 97.69% (Figure 1, Appendix C). On the other hand, species such as *Eucladium verticillatum*, *Brachythecium rivulare*, *Palustriella falcata*, *Dichodontium flavescens*, *D. pellucidum*, *Chiloscyphus polyanthos*, *Apopellia endiviifolia* and *Jungermannia atrovirens* had mean values of intensive agriculture within the catchment area lower than 5% and maxima lower than 15%. Considering the urban area within the catchment, most species preferred low levels, with rheophytes such as *Rhynchostegium riparioides, Cinclidotus fontinaloides, C. aquaticus, C. riparioides, Fontinalis antipyretica* and *Cratoneuron filicinum* peaking below 12% and then decreasing in abundance and finally disappearing at around 30% of urban area within the catchment. 

## 3. Discussion

Our results are in line with previous studies, confirming that different water chemistry factors associated with water quality influence the distribution and segregation of freshwater bryophytes [37,38]. Similarly, changes in land use within the catchment area, such as an increase in the share of intensive agriculture or urban area, which are most often associated with water pollution and eutrophication, also influence freshwater bryophytes [20].

The results of our study reveal that most of the studied freshwater bryophytes prefer natural catchments with clear, well-oxygenated water that has low nutrient levels, both nitrogen and phosphorus, as well as a low amount of organic matter. Similar results were obtained from the study of the freshwater bryophytes in the Tiber River Basin (Italy), where the majority of the species showed a general preference for fast-flowing, clear, cold and oxygenated water with a low nutrient load, especially low ammonia and orthophosphates [21]. 

Although most species in our study had their optima in water of good quality, differences in their responses to investigated ecological gradients revealed a set of species with very narrow ecological niches concerning this group of parameters. Species such as *Palustriella falcata*, *Eucladium verticillatum*, *Jungermannia atrovirens* and *Dichodontium flavescens* were mostly characteristic of the Dinaric Ecoregion and were recorded in pristine karstic watercourses with low nutrient content and low organic matter. Similar results were reported earlier for these species. In a study dealing with Natura 2000 petrifying sources in Belgium, the occurrence of *Eucladium verticillatum*, a tufa-forming species, was negatively correlated with ammonium and phosphate concentration, and the species preferred open habitats with lots of light [41]. *Palustriella falcata*, known as calcicole, is mostly linked to base-rich, neutro-alkaline, cold and turbulent oligotrophic streams with low conductivity [3,18,39], while *Jungermannia atrovirens* was reported in both oligotrophic and meso-oligotrophic waters [55]. *Dichodontium flavescens* was in our study almost exclusive to the Dinaric-Continental Subecoregion and restricted to clean, cold and turbulent karstic watercourses with almost completely natural catchment areas. Similarly, according to Dierßen [56], *Dichodontium flavescens* inhabits areas under no or only weak human impact.

Additionally, species such as *Cinclidotus aquaticus*, *Chiloscyphus polyanthos*, *Apopellia endiviifolia* and *Didymodon tophaceus* had somewhat wider niches, with optima in oligotrophic waters but tolerating elevated nutrient levels to some extent. However, their presence in higher abundance may be indicative of good water quality. *Cinclidotus aquaticus*, was previously reported as a species of clear, cold and turbulent oligotrophic waters and highlighted as a valid indicator of water quality [21], unlike *Apopellia endiviifolia* which was found across a broader spectrum of water quality, from oligotrophic [38,57] to eutrophic [21,41,58], tolerating high nitrate, ammonium and orthophosphate concentration.

All the above-mentioned species are basophilous and are either exclusive to or dominantly occur in karstic rivers of the Dinaric Ecoregion. These rivers flow over carbonate bedrock which influences water pH and alkalinity, as well as species assemblages where basophilous, i.e., acid-sensitive, taxa dominate. An important influence of geology, water pH and alkalinity on aquatic bryophytes has been already demonstrated on the European level [1,2,4,5,30,39,57] and beyond [6,17,40,59], and clear segregation of aquatic bryophytes along the alkalinity and pH gradient was recently demonstrated for Croatian bryophyte-dominated watercourses as well [22]. The latter study identified three communities characterized by different basophilous species which were mostly associated with karstic rivers of the Dinaric Ecoregion, and two communities in small rivers situated in the Pannonian Ecoregion, which were dominated by a high share of hygrophyte taxa inhabiting periodically flooded river margins. The influence of the water pH on aquatic bryophytes was evident in our study as well, although the investigated pH gradient was quite short. The majority of the species included in our study preferred near-neutral to basic water, with the mosses *Eucladium verticillatum*, *Didymodon tophaceues*, *Palustriella falcata, Brachythecium rivulare, Fissidens crassipes* and *Cinclidotus aquaticus*, as well as the liverworts *Apopellia endiviifolia* and *Chiloscyphus polyanthos*, being most strongly associated with higher water pH and having high frequencies and abundance in the watercourses of the Dinaric Ecoregion. This ecoregion is known to harbor greater diversity regarding freshwater bryophytes [15] and their communities [22] since its fast, cold montane and semi-montane karstic rivers with larger and more stable substrates provide more suitable habitats than the lowland rivers of the Pannonian Ecoregion, which are usually slow and warmer, with dominantly sandy and gravelly substrates. This was demonstrated in our study as well, with the highest number of species recorded in montane and mid-altitude small watercourses, followed by montane and mid-altitude medium and large watercourses of the Continental Subecoregion, which are both characterized by the dominance of large and medium substrates.

Widely distributed and frequent species such as *Fontinalis antipyretica*, *Rhynchostegium riparioides*, *Cratoneuron filicinum*, *Fissdens crassipes*, *Brachythecium rivulare*, *Cinclidotus riparius* and *C. fontinaloides* displayed in our research broad ecological tolerance regarding the investigated environmental gradients, with their maxima of ammonium, nitrate and total nitrogen concentrations exceeding the thresholds for good water chemistry status set for all water body types included in this analysis according to the Croatian Regulation on Water Quality Standard [60]. Furthermore, the same was observed in the case of their maxima for total phosphorus, except for *Brachythecium rivulare* and *Fontinalis antipyretica*. The maxima of the latter two species did exceed the values set for good status considering total phosphorous for all water body types of the Dinaric Ecoregion, which are naturally more oligotrophic. However, for all investigated Pannonian water body types, they exceeded the values set for very good status. Regarding the organic matter content, the COD maxima of *Fontinalis antipyretica*, *Rhynchostegium riparioides*, *Cratoneuron filicinum*, *Fissdens crassipes*, *Cinclidotus riparius* and *C. fontinaloides* again exceeded thresholds set for good status for all types in the study, while that of *Brachythecium rivulare* exceeded thresholds set for all investigated Dinaric types, as well as thresholds for very good status regarding this parameter for investigated Panonnian types. Nevertheless, all of these species had their optima in clean, oligotrophic, oxygenated and slightly basic water of low electrical conductivity, except for *Fontinalis antipyretica*, which preferred high values of the latter parameter. *Fontinalis antipyretica* and *Rhynchostegoium riparioides*, which are both widespread and the most common species in Croatia [15], are often found together occupying a wide range of freshwater habitats [18,39,61,62]. Their broad trophic range, and in general wide ecological behavior, have been emphasized by several authors [3,18,24,25,38], with *Fontinalis antipyretica* being more tolerant of eutrophication and elevated electrical conductivity [21,38]. However, its frequency was reported to increase with decreasing concentrations of nitrates and phosphates [21], as was the case with its abundance in our study. Interestingly, *Fontinalis antipyretica*, as well as *Fissides crassipes*, displayed distinctly different response curves in two hydrographic networks in France and Belgium [37], having a maximal frequency in oligotrophic water in one and tolerating the most polluted waters in the other, while their overall optima within the study were in eutrophic waters when data from both hydrographic networks were considered simultaneously. The authors suggested that such species may include several ecotypes with different trophic requirements within different hydrographic networks, which is possibly a result of microevolution, favoured by the fact that river basins are rarely interconnected. The observed differences in autecology between populations of the same species complicate the use of certain aquatic bryophytes as bioindicators of water quality on a large scale, and thus further research on their distribution and ecological responses, as well as their taxonomy, microevolution processes and ecophysiology, is still welcomed to elucidate the influence of environmental factors on the species that have so far shown contradictory behavior. 

Furthermore, studies encompassing larger geographic areas and gradients of water quality parameters could improve the knowledge of the autecology and ecological tolerance of species that have shown contradictory behavior in different studies. An example of contradictory findings being reported for the same species can be seen in the case of the common basophilous species, *Cratoneuron filicinum*. It was reported as a valid bioindicator of water quality with narrow ecological tolerance, preferring clear, turbulent waters, with temperature below 12 °C, low conductivity (below 300 μS/cm), and low concentration of nutrients (phosphates about 0.01 mg/L, a maximum concentration of ammonium 0.10 mg/L and nitrates 0.90 mg/L), in a study covering the Tiber River Basin [21], while tolerance to light to moderate eutrophication has been reported in several other studies as well [20,41,58]. In our study, which encompassed a larger and more diverse geographic area with many different hydrographic networks, a broader ecological behavior of this species was observed. It was more frequent in colder watercourses, but occurred in much warmer waters as well, with a maximum of 18.75 °C. Similarly, it preferred medium conductivity of about 420 μS/cm, while tolerating levels as high as 941 μS/cm. Regarding the nutrients, its optimum was in clean water of low trophy level, but the species persisted in waters with a great nutrient load as well (e.g., maximum concentration of total phosphorous 0.28 mgP/L, orthophosphates 0.09 mgP/L, total nitrogen 2.00 mgN/L and ammonium 0.86 mgN/L). 

*Riccia fluitans* and *Leptodyctium riparium* showed markedly different behavior from all the other species in our study, preferring neutral, warmer, hypereutrophic water with high electrical conductivity and organic matter content, which is in line with previous findings [18,21,23,26,37,63]. In our study, although these species displayed quite wide water quality niches, their abundance was the highest in eutrophic situations, with a prominent fall in abundance with an increase in water quality. Similarly, the frequency of these species was positively correlated with the concentration of phosphates and ammonia, as well as electrical conductivity, in a study conducted by Ceschin et al. [21] in Italy. While these authors referred to both species as indicators of eutrophication, others found that *Leptodyctium riparium* exhibited a broad ecological range [37], from oligotrophic streams to hypertrophic rivers, and did not appear as a reliable indicator, although its frequency increased under eutrophic conditions [38]. Noteworthy is that in our study, the optima of *Riccia fluitans* for total nitrogen and total phosphorous (8.9 mgN/L and 0.7 mgP/L, respectively) were considerably higher than those of *L. riparium* (4.9 mgN/L and 0.21 mgP/L, respectively), and the species favoured the sites with the least natural catchment areas with a large proportion of intensive agriculture. *Leptodyctium riparium* was also associated with a low proportion of natural area within the catchment, with an optimum of 33.57%. The case was similar in highly seasonal rivers in Bulgaria, where the species was characteristic for sites located in regions with increased intensive agriculture and watercourses with reduced flow and pronounced silting, as well as elevated total nitrogen concentration [20]. Both species had a higher frequency in the Pannonian than in the Dinaric Ecoregion of Croatia, which is clearly related to the characteristics of its water bodies; these are more frequently slow and eutrophic lowland streams, rivers and canals with unstable sediment [64], which are known as less suitable habitats for bryophytes and support only modest diversity [10,15,65]. Here, the aquatic form of *Riccia fluitans* was recorded floating mostly in stagnant waters of hypereutrophic artificial canals, while *Leptodyctium riparium* was growing on rarely present large stable rocks, dead wood, periodically submerged tree bases and margins of the watercourses, having the highest frequency in lowland small watercourses, followed by lowland medium and large watercourses. These watercourses naturally have higher trophic status and the vast majority of them are additionally subjected to substantial changes in land use associated with high nutrient input, as well as hydromorphological degradation [66], which are known to reduce habitat quality for bryophytes, resulting in reduced cover, diversity and changes in community structure [14,17,20,22]. Thus, as expected, freshwater bryophytes which occur in higher frequency within this region are those which can tolerate poorer water-quality and inhabit less-natural catchment areas, such as *Leptodictyum riparium*, *Cratoneuron filicinum, Cinclidotus riparius*, *Rhynchostegium riparioides, Fontinalis antipyretica* and *Fissidens crassipes*. 

As the data used in this study were gathered in the course of the national macrophyte monitoring conducted for the purpose of assessing the ecological status of waterbodies as required by the WFD, we want to emphasize the importance of its implementation, as it encouraged research into freshwater bryophytes and their ecology on a national [15,22,67,68,69,70,71,72] and European level [1,2,4,5,14,28,29,50,51,53] by including this group as a part of macrophyte vegetation. Our research is the first into the ecology of the aquatic bryophytes in Croatia, exploring the ecological responses of the most frequent species and determining the influence of different environmental variables on their occurrence. These results make a solid base from which the bioindication potential of these particular species can be inferred, based on their optima and niche width, and a starting point crucial for the improvement and adjustment of the national methodology regarding the bryophytes as an integrative part of macrophyte vegetation. It should be noted that WFD takes a more holistic approach than traditional monitoring practices and requires the assessment of the ecological status, which must be determined type-specifically. Namely, for each type of water body recognized by the national typology, reference conditions should be identified, and degradation has to be quantified as the deviation in species composition and abundance from those that would be present at reference conditions [73]. Having this in mind, our results are a good starting point, because they add information on species currently not included and additional information on ecological responses for a few species already included in the Croatian methodology. However, particular species scores and the inclusion of new species into this methodology should be derived considering the type-specific reference conditions to meet the requirements of the WFD.

## 4. Materials and Methods

### 4.1. Study Area

A total of 648 sampling sites on 382 different watercourses were surveyed during vegetation seasons from 2016 to 2021. Surveys were carried out within the national surface water monitoring scheme, which is conducted to assess the ecological status of water bodies as required by the Water Framework Directive (WFD). The sampling sites were preselected to encompass the heterogeneity of different water body types recognized by the typology developed as a basis for the monitoring of surface waters [60], with all water body types represented proportionally and fulfilling the requirements of the stratified sampling. This typology recognizes two hydrological and biogeographical regions in Croatia—the Pannonian and the Dinaric Ecoregion, the latter being subdivided into Continental and Mediterranean subecoregions (Figure 1). 

The Pannonian Ecoregion refers to the continental, lowland part of the country, largely converted into agricultural areas. The geological bedrock is dominantly siliceous, while the climate is temperate, without a dry season, with warm summers (Cfb), becoming hotter towards the east (Cfa) [74]. The Dinaric Ecoregion refers to the central and western part of the country, with a dominant karstic landscape, developed on limestone and dolomite bedrock. It is divided into the Continental Subecoregion, characterized by a temperate climate (Cfb), and the Mediterranean Subecoregion with mostly Mediterranean climate, with dry and hot summer months (Csa) [74]. The Pannonian watercourses and the majority of the watercourses of the Dinaric-Continental Subecoregion belong to the Black Sea Basin, while the watercourses of the Dinaric-Mediterranean Subecoregion belong to the Adriatic Sea Basin.

### 4.2. Vegetation Data Sampling

Macrophyte vegetation was surveyed from June to September, during the main vegetation period and the lowest water discharge levels. Following the national methodology for macrophyte sampling [60], watercourses were surveyed along 100 m-long transects from the banks and by zigzagging across the channel if the water depth was low enough. The vegetation survey included all macrophyte representatives (bryophytes, vascular plants and macroalgae), and the cover and abundance of each species was assessed using the standard Central European phytocoenological methodology, i.e., extended Braun–Blanquet scale (r = one individual, + = up to 5 individuals, 1 = up to 50 individuals, 2m = over 50 individuals but coverage < 5%, 2a = coverage 5–15%, 2b = coverage 15–25%, 3 = 25–50%; 4 = coverage 50–75%; 5 = coverage over 75%) [75,76,77], which was further transformed to the van der Maarel scale from 1 to 9 [78] (Appendix D). To investigate the ecological preferences and autecology of freshwater bryophytes, further analysis included only bryophytes with ≥5 occurrences that fall into categories of greater water affinity according to Dierßen [56]. These were collected from various substrates (e.g., rocks, boulders, pebbles, xylal) within the riverbed, as well as from the periodically flooded river margins. Voucher specimens were deposited at the Herbarium collection ZA [79]. The nomenclature follows Hodgetts et al. [80].

### 4.3. Environmental Data Sampling and Acquisition

All localities were also sampled for basic water physicochemical and chemical analysis once a month throughout the year. Water temperature, electrical conductivity, pH and dissolved oxygen were measured in situ with a Hach HQ40D Portable Multi Meter under standard conditions. Furthermore, water samples were collected and analyzed in an accredited laboratory (Central Water Management Laboratory, Zagreb) for total alkalinity, total suspended solids, biochemical oxygen demand and chemical oxygen demand, as well as for nitrogen and phosphorus compounds (ammonium, nitrites, nitrates, total nitrogen, orthophosphates and total phosphorus) (Table 1). The land use in the catchment area of each sampling site was obtained from the database of Hrvatske vode—the legal entity for water management. Here, four distinct categories are recognized and calculated from the CORINE land cover dataset [81]—natural area, urban area, and intensive and extensive agricultural land (Table 1).

### 4.4. Data Analysis

Data analysis included rheophyte, hydrophyte, amphyphyte and hygrophyte species [56] occurring in at least five of the surveyed localities (a total of 21 species from 182 localities) (Table S1) matched with 18 environmental variables (Table S2). This data set included thirteen different types of watercourses according to the current national typology (Appendix A, Table A1) and was used to compile a frequency table from the species occurrence within each type (Appendix A, Table A2). Basic descriptive statistics (min, max, mean, SE, SD and median) of all environmental variables were calculated for the species (Appendix C) in Past 4.9 software [82] and their distribution along the gradient of each environmental variable was shown with box-plot graphs created in SPSS software (Figure 1). Furthermore, the descriptive statistic was calculated for all environmental variables for the Pannonian and Dinaric Ecoregion (Table A6, Appendix E), as well as for the Dinaric-Continental and Dinaric-Mediterranean subecoregion (Table A7, Appendix E).

To assess the relationship between the environmental variables and patterns in freshwater bryophyte species composition, a direct ordination method, canonical correspondence analysis (CCA), was used. After removing the outliers, vegetation and environmental data from 176 localities were included in the analysis. CCA was selected because the response data were compositional with a gradient longer than 4.2 SD units, meaning that analysis based on a unimodal, rather than the linear model, is preferred [83]. A step-forward selection procedure in CANOCO 5 [83,84] was used to identify the most-contributing subset of environmental predictors influencing the freshwater bryophytes. Eight variables with the highest conditional effect and with a 5% significance cut level (*p* < 0.05; Monte Carlo test, 499 permutations) were included. Prior to the analysis, species abundance values were square-rooted and rare species downweighted.

Generalized additive models (GAM) were employed to model the probability of occurrence of individual bryophyte species as a function of eight environmental variables highlighted in the CCA analysis. GAMs were selected as an efficient tool in ecology since they do not require an assumption about the shape of species response along the environmental gradient [83,84,85,86]. We used Poisson distribution with log link function and df = 2 in fitting the species response curves and Akaike Information Criterion (AIC) available in CANOCO 5 [83,84] to select the best model. AIC considers not only the goodness of fit but also selects the most parsimonious model. Species for which no candidate model had an AIC value lower than the null model were automatically detected and removed by this procedure. Furthermore, only statistically significant models (*p* < 0.05) were retained and shown in graphs (Table S3).

## 5. Conclusions

The present study revealed that freshwater bryophytes and their assemblages were segregated along the gradients of the water chemistry and the proportion of natural and urban area within the catchment. The two latter variables represent the degree of combined stress, because they are often related to the extent of eutrophication, and pollution in general as well as hydromorphological degradation, of the watercourses. Furthermore, the ecological responses of individual species were examined to determine their optima, degree of tolerance and bioindication potential regarding the studied variables. The results showed that most of the investigated species preferred natural, clean, well-oxygenated, oligotrophic watercourses, with low organic matter content and electrical conductivity. However, the widely distributed and most frequent species, such as *Fontinalis antipyretica*, *Rhynchostegium riparioides*, *Cratoneuron filicinum*, *Fissidens crassipes*, *Cinclidotus fontinaloides* and *C. riprarius*, showed wide ecological tolerance to studied water chemistry variables and are thus not reliable bioindicators concerning these variables. On the other hand, species such as *Palustriella falcata*, *Eucladium verticillatum*, *Dichodontium flavescens* and *Jungermannia atrovirens* had narrow ecological niches and were restricted to pristine watercourses. *Riccia fluitans* and *Leptodyctium riparium* were the most obviously separated from the rest of the species, being the most tolerant to poor water quality. These species had wide ecological ranges, but preferred neutral, hypereutrophic waters with high nutrient and organic content, as well as electrical conductivity. Furthermore, they were frequently associated with a higher share of intensive agriculture and a low share of natural land within the catchment. 

The ecological responses of several species obtained from our study do not perfectly correlate with previous findings. This might be a result of different methodological approaches concerning data collection and analysis, differences in the gradient length encompassed within each study, or the existence of different ecotypes of particular freshwater bryophytes, which display different ecological behavior in different geographical areas. Nevertheless, our study covered a considerable geographic area, included many different types of watercourses, from ground-fed streams to eutrophic large rivers, and thus encompassed substantially long gradients of the environmental parameters investigated. This has provided new data on the ecology and bioindication potential of freshwater bryophytes, contributing to the existing body of knowledge on both subjects.

## Figures and Tables

**Table 1:** Environmental variables and abbreviations used.

	Environmental Variable	Abbreviation
Water physicochemical parameters	Water temperature	T (°C)
	Water pH	pH
	Electrical conductivity	EC (μS/cm)
	Total suspended solids	TSS (mg/L)
	Dissolved oxygen	DO (mgO₂/L)
	Total alkalinity	ALK (mgCaCO₃/L)
	Biochemical oxygen demand	BOD (mgO₂/L)
	Chemical oxygen demand	COD (mgO_2_/L)
Water chemical parameters	Ammonium	NH₄^+^ (mgN/L)
	Nitrites	NO_2_^−^ (mgN/L)
	Nitrates	NO_3_^−^ (mgN/L)
	Total nitrogen	N_tot_ (mgN/L)
	Orthophosphates	PO_4_^3−^ (mgP/L)
	Total phosphorus	P_tot_ (mgP/L)
Land use within the catchment area	Natural area	NAT (%)
	Intensive agriculture	IAG (%)
	Extensive agriculture	EAG (%)
	Urban area	URB (%)

## Data Availability

The data presented in this study are available on request from the corresponding author.

## Supplementary Materials

The following supporting information can be downloaded at: https://www.mdpi.com/article/10.3390/plants11243451/s1, Table S1: Cover and abundance of the species at each surveyed locality with coordinates of localities in WGS84; Table S2: Yearly mean values of 18 environmental parameters for all surveyed localities, Table S3: Results of the generalized additive models (GAMs).

## Appendix A

**Table A1:** Types of surveyed watercourses according to the national methodology with main characteristics. ALT–altitude, CA–catchment area.

Watercourse Type		No. of Sites	ALT (m a.s.l.)	CA (km^2^)	Substrate Size
Pannonian Ecoregion					
1.	Montane and mid-altitude small watercourses	7	200–>500	10–100	large, medium
2.	Lowland small watercourses	33	<200	10–100	small
3.	Lowland medium and large watercourses	15	<200	100–10,000	small, medium
**Dinaric-Continental Ecoregion**					
4.	Montane and mid-altitude small watercourses	24	200–>500	10–100	large, medium
5.	Montane and mid-altitude medium and large watercourses	17	200–>500	100–10,000	large, medium, small
6.	Lowland medium and large watercourses	10	<200	100–10,000	small, medium
7.	Montane and mid-altitude intermittent watercourses	8	200–>500	10–100	large, medium
**Dinaric-Mediterranean Ecoregion**					
8.	Lowland and mid-altitude small watercourses	17	0–500	10–100	medium, large
9.	Mid-altitude medium and large watercourses	12	200–500	100–10,000	medium, large
10.	Lowland medium and large watercourses	11	<200	100–10,000	medium, large
11.	Lowland and mid-altitude watercourses running through karst field	4	<200–500	10–1000	small
12.	Intermittent rivers of Mediterranean Subecoregion	14	<200–500	10–1000	large, small
**Artificial canals**					
13.	Artifical waterbodies	10	<200–500	10–10,000	small, medium, large

**Table A2:** Species frequencies across different types of watercourses. For abbreviations of species’ names see Figure 2, and for types see Table A1 of Appendix A.

Watercourse Type	1.	2.	3.	4.	5.	6.	7.	8.	9.	10.	11.	12.	13.
No. of sites	7	33	15	24	17	10	8	17	12	11	4	14	10
Fon ant	29	18	40	42	59	70	75	65	92	55	75	43	30
Rhy rip	57	33	27	58	71	70	38	59	50	45	.	64	10
Lep rip	14	76	73	42	18	10	50	18	17	18	.	.	.
Cra fil	29	24	7	58	53	50	38	53	33	18	.	36	.
Cin fon	.	3	13	33	76	40	25	29	8	18	.	50	30
Fis cra	.	6	27	33	35	50	13	35	8	18	.	21	.
Cin rip	.	.	40	25	59	60	25	24	.	.	.	14	.
Cin aqu	.	3	.	21	47	60	13	41	33	.	.	14	10
Apo end	.	6	.	25	41	20	13	41	25	9	25	14	.
Mar pol	.	15	7	38	24	20	13	12	.	.	.	.	.
Chi pol	.	12	.	8	47	10	.	35	17	.	.	.	.
Pty pse	.	21	7	4	18	10	13	6	8	18	.	7	.
Bra riv	.	6	.	17	29	20	.	6	.	.	.	.	.
Pal fal	.	3	7	.	24	10	.	.	25	.	.	.	.
Did top	.	.	.	.	6	10	.	18	8	18	.	14	.
Chi pal	14	9	.	4	.	10	.	6	.	.	.	.	.
Ric flu	.	.	7	.	6	.	.	.	.	.	25	.	40
Jun atr	.	.	.	8	18	.	13	.	.	9	.	.	.
Euc ver	.	.	.	4	12	.	13	6	.	18	.	.	.
Dic fla	.	3	.	8	12	.	.	.	.	.	.	.	.
Dic pel	.	6	.	4	12	.	.	.	.	.	.	.	.

## Appendix B

**Table A3:** Results of the forward selection procedure of environmental variables for the canonical correspondence analysis, CCA. For abbreviations of environmental variables see Table 1.

Variable	Explains % (Simple Effect)	Explains % (Conditional Effect)	Contribution %	Pseudo-F	*p*
N_tot_	4.8	4.8	23.3	8.9	0.002
NAT	4.6	2.9	13.8	5.4	0.002
COD	3.9	1.5	7.1	2.8	0.004
EC	2.0	1.2	5.8	2.3	0.008
pH	1.1	1.1	5.5	2.2	0.008
P_tot_	4.5	1.0	5.2	2.1	0.012
URB	1.5	1.0	4.9	2.0	0.022
DO	2.3	1.1	4.8	2.0	0.030

## Appendix C

**Table A4:** Descriptive statistics of all environmental variables for 21 freshwater bryophytes. For abbreviations of species’ names see Figure 2, and for environmental variables see Table 1.

		Apo end	Bra riv	Chi pal	Chi pol	Cin aqu	Cin fon	Cin rip	Cra fil	Dic fla	Dic pel	Did top	Euc ver	Fis cra	Fon ant	Jun atr	Lep rip	Mar pol	Pal fal	Pty pse	Rhy rip	Ric flu
**T (°C)**	**Min**	8.88	9.13	10.45	8.88	7.85	8.42	7.00	8.70	9.13	10.94	9.80	9.57	7.00	7.00	9.57	7.00	9.13	7.85	8.88	7.00	12.38
	**Max**	17.58	17.15	14.29	15.30	16.06	16.73	16.22	18.75	13.21	13.21	17.85	15.66	18.63	19.30	15.66	20.17	18.75	15.65	17.85	18.75	17.19
	**Mean**	12.14	11.73	12.15	11.33	11.59	12.22	12.37	12.13	11.13	11.81	13.64	11.98	12.74	12.73	11.66	12.86	13.35	11.21	12.43	12.38	14.54
	**SE**	0.38	0.62	0.53	0.28	0.36	0.34	0.37	0.32	0.74	0.43	0.90	0.77	0.44	0.24	0.80	0.29	0.60	0.71	0.57	0.26	0.68
	**SD**	2.18	2.34	1.41	1.37	2.15	2.35	2.22	2.50	1.65	0.95	2.84	2.03	2.70	2.27	2.11	2.31	2.94	2.25	2.49	2.43	1.80
	**Med**	11.87	10.98	12.36	11.01	10.94	11.85	12.51	11.32	10.94	11.48	14.28	12.16	12.71	12.49	10.94	12.73	12.84	10.87	12.36	12.33	14.92
**pH**	**Min**	7.59	7.48	7.03	7.48	7.48	7.13	7.60	7.03	7.87	7.66	7.58	7.78	7.56	7.13	7.78	7.03	7.56	7.73	7.13	7.13	7.06
	**Max**	8.28	8.35	8.28	8.28	8.35	8.35	8.35	8.35	8.28	8.28	8.20	8.20	8.35	8.38	8.28	8.28	8.35	8.28	8.28	8.35	7.87
	**Mean**	7.97	8.07	7.70	7.94	7.96	7.93	8.01	7.96	8.14	8.04	7.94	8.00	8.02	7.95	8.05	7.89	8.00	8.09	7.89	7.95	7.55
	**SE**	0.04	0.06	0.17	0.05	0.04	0.04	0.03	0.03	0.07	0.12	0.07	0.07	0.04	0.03	0.07	0.03	0.04	0.06	0.06	0.03	0.10
	**SD**	0.22	0.23	0.46	0.24	0.22	0.26	0.20	0.25	0.16	0.27	0.23	0.17	0.24	0.24	0.19	0.22	0.22	0.20	0.27	0.25	0.26
	**Med**	8.02	8.14	7.84	7.92	7.96	7.91	8.05	8.00	8.18	8.13	7.97	7.99	8.10	7.95	8.13	7.92	8.02	8.17	7.87	7.96	7.53
**EC (μS/cm)**	**Min**	262.08	147.04	227.33	80.34	228.58	189.33	147.04	147.04	147.04	147.04	280.10	280.10	147.04	80.34	147.04	80.34	189.36	262.08	189.36	121.05	306.33
	**Max**	941.71	504.75	458.82	719.00	1073.0	802.91	802.91	941.71	295.42	302.20	941.71	366.75	941.71	1073.0	366.75	641.75	641.75	435.57	583.91	948.33	923.50
	**Mean**	412.45	335.01	335.57	374.50	410.76	400.31	365.58	419.20	234.66	240.17	483.75	331.49	418.57	449.88	292.20	460.15	401.99	335.74	343.62	420.05	700.75
	**SE**	21.55	29.32	28.97	30.85	30.39	19.51	21.33	19.91	28.44	30.38	62.98	12.07	23.55	17.98	28.37	15.51	25.72	15.41	22.43	18.33	97.80
	**SD**	123.82	109.71	76.65	147.94	179.77	135.18	127.96	156.74	63.59	67.94	199.17	31.95	145.16	169.59	75.07	124.08	126.02	48.74	97.76	171.00	258.76
	**Med**	366.75	325.55	345.00	358.50	373.00	367.83	351.97	389.04	262.08	279.42	417.89	340.14	390.84	404.50	296.17	469.18	403.47	330.75	345.00	391.90	831.75
**TSS (mg/L)**	**Min**	1.00	1.00	1.00	0.85	0.85	1.00	1.00	0.85	1.00	1.00	1.64	1.00	1.00	0.95	1.00	1.00	1.00	0.85	1.00	0.95	1.00
	**Max**	17.12	16.00	12.20	20.92	8.92	23.00	27.33	18.90	8.59	20.92	6.53	6.53	27.33	49.50	1.64	49.80	21.54	3.50	20.92	47.94	40.17
	**Mean**	2.67	3.75	5.50	2.94	2.25	2.83	4.29	3.23	2.52	6.50	3.33	2.06	3.91	4.79	1.09	10.22	4.66	1.61	4.78	4.05	14.42
	**SE**	0.66	1.20	1.77	0.98	0.31	0.50	0.93	0.50	1.52	3.89	0.50	0.77	0.85	0.86	0.09	1.34	1.17	0.34	1.20	0.67	5.03
	**SD**	3.81	4.48	4.69	4.68	1.85	3.49	5.60	3.92	3.39	8.70	1.59	2.03	5.22	8.09	0.24	10.70	5.74	1.07	5.23	6.26	13.32
	**Med**	1.00	1.70	5.75	1.00	1.30	1.82	2.11	1.67	1.00	1.00	2.68	1.00	2.09	2.00	1.00	7.07	2.06	1.00	2.02	2.08	12.17
**DO (mgO** **₂** **/L)**	**Min**	8.86	9.42	9.25	9.69	9.66	7.95	8.86	8.29	10.78	10.16	7.51	8.86	8.86	5.57	8.86	4.84	9.11	10.13	6.15	6.15	5.25
	**Max**	12.45	12.22	11.66	12.45	12.45	12.45	14.25	12.55	12.22	12.22	11.84	11.68	12.55	14.25	12.22	14.25	12.22	12.45	14.25	14.25	9.34
	**Mean**	10.76	11.06	10.26	11.07	11.07	10.63	11.04	10.65	11.58	11.29	10.13	10.50	10.78	10.64	10.83	9.99	10.80	11.40	10.32	10.60	7.74
	**SE**	0.15	0.21	0.42	0.15	0.11	0.15	0.21	0.12	0.23	0.37	0.38	0.38	0.16	0.12	0.49	0.22	0.19	0.25	0.41	0.12	0.57
	**SD**	0.84	0.77	1.10	0.74	0.66	1.06	1.23	0.93	0.52	0.82	1.21	1.02	1.02	1.12	1.29	1.74	0.95	0.78	1.78	1.13	1.50
	**Med**	10.92	11.26	9.54	11.10	11.06	10.85	11.09	10.78	11.68	11.48	10.16	10.93	10.92	10.85	11.48	10.17	10.99	11.44	10.38	10.75	7.93
**ALK (mgCaCO** **₃** **/L)**	**Min**	98.67	77.03	110.67	48.00	126.92	90.33	77.03	77.03	77.03	76.19	167.64	167.64	77.03	48.00	77.03	48.00	91.45	152.75	76.19	59.82	171.58
	**Max**	281.67	255.00	229.17	272.55	252.10	344.62	288.60	308.41	161.50	175.70	282.40	208.04	288.60	344.62	208.04	343.33	308.41	200.14	308.41	344.62	446.67
	**Mean**	202.95	180.30	187.63	180.76	188.77	203.13	185.85	207.25	128.55	116.38	218.63	185.50	207.53	205.16	160.72	228.16	209.49	179.42	184.85	196.81	306.08
	**SE**	6.93	15.26	15.54	12.12	5.31	7.86	8.38	6.48	18.29	21.61	12.54	5.35	8.10	5.16	15.52	8.19	11.22	4.54	13.11	6.17	43.45
	**SD**	39.80	57.11	41.12	58.10	31.39	54.47	50.29	51.05	40.90	48.32	39.65	14.15	49.94	48.68	41.07	65.48	54.98	14.37	57.16	57.55	114.95
	**Med**	194.57	182.80	198.00	190.50	187.78	195.26	186.55	197.04	152.75	91.45	210.23	185.83	197.15	199.20	167.64	230.38	220.50	181.21	181.92	194.57	290.58
**BOD (mgO** **₂** **/L)**	**Min**	0.30	0.30	0.30	0.30	0.30	0.14	0.52	0.25	0.62	0.69	0.30	0.30	0.30	0.30	0.62	0.30	0.30	0.30	0.30	0.14	0.70
	**Max**	2.70	2.90	2.71	2.64	2.64	2.80	3.32	7.33	1.58	2.60	1.71	2.63	3.44	3.60	2.63	6.90	2.90	1.43	3.32	9.78	5.10
	**Mean**	1.00	1.18	1.42	0.96	0.97	1.05	1.32	1.24	1.02	1.30	1.16	1.27	1.17	1.20	1.09	2.08	1.19	0.83	1.53	1.37	3.62
	**SE**	0.11	0.20	0.36	0.13	0.09	0.07	0.12	0.14	0.18	0.36	0.14	0.29	0.11	0.09	0.27	0.15	0.15	0.12	0.20	0.13	0.62
	**SD**	0.62	0.75	0.94	0.64	0.55	0.51	0.73	1.10	0.40	0.81	0.45	0.76	0.71	0.83	0.70	1.19	0.71	0.39	0.88	1.22	1.63
	**Med**	0.84	0.90	1.84	0.80	0.87	1.01	1.09	0.94	0.84	0.84	1.33	1.18	0.93	1.07	0.84	1.75	1.09	0.79	1.18	1.10	4.39
**COD (mgO_2_/L)**	**Min**	0.25	0.25	0.49	0.25	0.25	0.49	0.77	0.25	0.78	1.77	0.50	0.49	0.25	0.25	0.89	0.29	0.25	0.66	0.72	0.25	1.27
	**Max**	4.36	4.36	10.67	5.51	4.30	10.68	6.95	10.67	4.97	5.51	10.68	2.52	10.68	10.68	2.34	13.11	4.97	2.26	10.67	10.83	7.55
	**Mean**	1.30	1.65	4.06	1.89	1.32	1.59	2.13	1.82	2.26	3.30	2.66	1.19	2.12	2.03	1.46	3.78	1.98	1.32	3.19	2.19	4.58
	**SE**	0.15	0.32	1.35	0.32	0.13	0.22	0.26	0.22	0.72	0.80	0.93	0.26	0.31	0.20	0.18	0.35	0.31	0.19	0.61	0.25	0.95
	**SD**	0.87	1.18	3.58	1.54	0.79	1.53	1.56	1.75	1.62	1.80	2.95	0.68	1.89	1.92	0.48	2.76	1.52	0.60	2.64	2.30	2.51
	**Med**	1.27	1.33	3.70	1.53	1.10	1.30	1.74	1.37	1.77	2.34	1.89	1.04	1.74	1.48	1.42	3.21	1.60	1.26	2.05	1.40	5.50
**NH** **₄** **+ (mgN/L)**	**Min**	0.001	0.004	0.004	0.004	0.003	0.002	0.003	0.002	0.004	0.004	0.001	0.001	0.002	0.001	0.002	0.001	0.002	0.001	0.002	0.001	0.009
	**Max**	0.221	0.221	0.361	0.313	0.149	0.665	0.665	0.862	0.004	0.313	0.044	0.009	0.665	0.498	0.009	1.697	0.221	0.017	0.361	0.862	0.212
	**Mean**	0.025	0.034	0.094	0.023	0.019	0.041	0.056	0.047	0.004	0.066	0.013	0.005	0.047	0.049	0.005	0.169	0.042	0.006	0.057	0.057	0.103
	**SE**	0.008	0.017	0.050	0.013	0.005	0.016	0.021	0.015	0.000	0.062	0.004	0.001	0.019	0.009	0.001	0.041	0.012	0.002	0.025	0.014	0.032
	**SD**	0.046	0.064	0.133	0.064	0.028	0.109	0.126	0.121	0.000	0.138	0.013	0.003	0.120	0.089	0.002	0.327	0.058	0.005	0.110	0.131	0.079
	**Med**	0.006	0.005	0.050	0.006	0.010	0.009	0.010	0.013	0.004	0.004	0.007	0.005	0.008	0.013	0.004	0.067	0.013	0.004	0.006	0.010	0.099
**NO_2_^−^ (mgN/L)**	**Min**	0.000	0.000	0.001	0.000	0.001	0.001	0.001	0.000	0.001	0.001	0.001	0.001	0.001	0.000	0.001	0.001	0.001	0.001	0.001	0.000	0.001
	**Max**	0.050	0.025	0.021	0.043	0.019	0.215	0.215	0.061	0.004	0.006	0.062	0.006	0.215	0.106	0.004	0.215	0.061	0.006	0.062	0.215	0.296
	**Mean**	0.008	0.004	0.008	0.005	0.004	0.011	0.011	0.008	0.002	0.003	0.013	0.002	0.014	0.009	0.002	0.023	0.012	0.003	0.008	0.010	0.052
	**SE**	0.002	0.002	0.003	0.002	0.001	0.005	0.006	0.002	0.001	0.001	0.007	0.001	0.006	0.002	0.001	0.005	0.003	0.000	0.003	0.003	0.041
	**SD**	0.013	0.006	0.007	0.009	0.005	0.034	0.036	0.012	0.001	0.002	0.021	0.002	0.036	0.017	0.002	0.036	0.016	0.001	0.014	0.027	0.107
	**Med**	0.002	0.002	0.007	0.002	0.002	0.003	0.003	0.003	0.002	0.002	0.005	0.001	0.003	0.004	0.001	0.010	0.004	0.003	0.004	0.003	0.013
**NO_3_^−^ (mgN/L)**	**Min**	0.060	0.196	0.184	0.060	0.179	0.092	0.092	0.060	0.325	0.325	0.060	0.092	0.060	0.092	0.092	0.184	0.225	0.301	0.092	0.092	0.075
	**Max**	1.098	1.594	0.603	1.212	1.594	1.594	1.594	1.594	0.643	1.212	0.909	0.695	1.594	1.472	0.695	4.442	1.350	0.802	1.212	4.442	3.142
	**Mean**	0.512	0.683	0.385	0.518	0.571	0.611	0.632	0.556	0.516	0.613	0.399	0.372	0.634	0.554	0.441	0.920	0.731	0.549	0.477	0.612	0.655
	**SE**	0.051	0.098	0.062	0.061	0.047	0.045	0.051	0.041	0.063	0.157	0.079	0.087	0.056	0.033	0.082	0.100	0.070	0.051	0.066	0.055	0.418
	**SD**	0.289	0.365	0.163	0.294	0.277	0.313	0.309	0.326	0.141	0.351	0.251	0.229	0.344	0.313	0.216	0.798	0.342	0.163	0.286	0.517	1.106
	**Med**	0.540	0.573	0.331	0.424	0.548	0.574	0.605	0.536	0.598	0.523	0.323	0.309	0.600	0.497	0.408	0.725	0.669	0.600	0.342	0.517	0.236
**Ntot (mgN/L)**	**Min**	0.125	0.305	0.275	0.278	0.275	0.158	0.158	0.125	0.489	0.489	0.275	0.158	0.158	0.158	0.158	0.389	0.275	0.393	0.158	0.158	0.478
	**Max**	1.385	2.081	1.159	1.606	1.765	2.243	2.243	2.000	0.786	1.606	1.257	1.150	2.243	2.458	0.786	5.176	2.638	1.150	1.606	5.176	8.900
	**Mean**	0.713	0.937	0.782	0.717	0.765	0.820	0.895	0.791	0.636	0.801	0.793	0.653	0.891	0.789	0.617	1.499	1.040	0.711	0.807	0.886	1.938
	**SE**	0.060	0.137	0.131	0.071	0.055	0.060	0.068	0.052	0.053	0.205	0.096	0.128	0.070	0.047	0.081	0.128	0.126	0.070	0.078	0.067	1.164
	**SD**	0.338	0.512	0.346	0.339	0.327	0.415	0.408	0.408	0.118	0.458	0.302	0.340	0.431	0.442	0.215	1.015	0.615	0.221	0.339	0.625	3.080
	**Med**	0.692	0.711	0.853	0.653	0.716	0.728	0.812	0.711	0.645	0.653	0.782	0.691	0.790	0.716	0.691	1.223	0.771	0.730	0.755	0.774	0.730
**PO_4_^3−^ (mgP/L)**	**Min**	0.001	0.002	0.003	0.001	0.001	0.001	0.001	0.001	0.004	0.006	0.001	0.001	0.001	0.001	0.002	0.003	0.001	0.002	0.001	0.001	0.003
	**Max**	0.020	0.040	0.026	0.026	0.027	0.179	0.179	0.089	0.010	0.026	0.029	0.003	0.179	0.095	0.007	0.188	0.070	0.009	0.038	0.179	0.473
	**Mean**	0.005	0.008	0.011	0.006	0.006	0.009	0.014	0.009	0.006	0.011	0.005	0.002	0.012	0.011	0.004	0.035	0.015	0.005	0.012	0.011	0.110
	**SE**	0.001	0.003	0.003	0.001	0.001	0.004	0.005	0.002	0.001	0.004	0.003	0.000	0.005	0.002	0.001	0.005	0.004	0.001	0.003	0.002	0.062
	**SD**	0.005	0.010	0.009	0.005	0.005	0.026	0.030	0.015	0.002	0.009	0.009	0.001	0.030	0.015	0.002	0.043	0.017	0.002	0.013	0.022	0.163
	**Med**	0.004	0.005	0.010	0.004	0.004	0.004	0.006	0.005	0.006	0.007	0.003	0.003	0.005	0.005	0.003	0.017	0.007	0.004	0.004	0.005	0.077
**Ptot (mgP/L)**	**Min**	0.001	0.005	0.005	0.001	0.001	0.005	0.002	0.001	0.008	0.010	0.002	0.005	0.002	0.001	0.005	0.004	0.002	0.005	0.002	0.001	0.001
	**Max**	0.064	0.079	0.070	0.091	0.045	0.271	0.271	0.280	0.038	0.091	0.042	0.025	0.271	0.175	0.025	1.067	0.223	0.034	0.093	0.271	0.699
	**Mean**	0.018	0.027	0.039	0.016	0.018	0.027	0.036	0.028	0.020	0.033	0.011	0.010	0.030	0.030	0.012	0.094	0.043	0.013	0.034	0.035	0.177
	**SE**	0.003	0.006	0.011	0.004	0.002	0.006	0.008	0.006	0.006	0.015	0.004	0.003	0.008	0.004	0.003	0.018	0.010	0.003	0.007	0.005	0.090
	**SD**	0.017	0.022	0.030	0.019	0.012	0.038	0.050	0.045	0.014	0.034	0.014	0.008	0.048	0.034	0.007	0.141	0.050	0.010	0.031	0.045	0.239
	**Med**	0.011	0.021	0.054	0.010	0.013	0.022	0.023	0.017	0.012	0.015	0.006	0.006	0.016	0.019	0.010	0.064	0.032	0.010	0.025	0.022	0.127
**NAT (%)**	**Min**	4.22	61.05	35.37	69.16	52.06	21.34	38.85	0.00	92.36	78.02	21.34	64.71	0.00	0.00	64.71	19.32	0.00	72.60	35.37	0.00	0.00
	**Max**	99.78	100.00	93.88	100.00	99.39	99.78	99.39	100.00	99.39	98.51	88.82	99.78	99.78	100.00	99.78	100.00	100.00	99.39	99.78	100.00	83.99
	**Mean**	80.60	88.47	70.98	88.04	82.32	78.92	76.22	78.76	96.55	91.45	65.64	80.30	72.89	73.10	85.30	59.87	77.75	85.90	72.82	75.65	34.91
	**SE**	3.09	3.09	8.28	1.68	2.04	2.51	2.88	2.40	1.35	3.71	6.44	5.06	3.72	1.91	6.00	2.62	4.57	2.57	4.21	2.16	12.87
	**SD**	17.76	11.57	21.90	8.07	12.06	17.39	17.25	18.88	3.01	8.31	20.36	13.39	22.94	18.03	15.87	20.93	22.40	8.13	18.33	20.15	34.05
	**Med**	86.90	91.33	74.62	89.08	83.69	83.69	81.08	85.47	98.05	92.36	70.57	77.82	79.09	77.32	92.36	59.81	86.00	85.99	76.14	81.24	15.88
**IAG (%)**	**Min**	0.00	0.00	0.00	0.00	0.00	0.00	0.00	0.00	0.00	0.00	0.00	0.00	0.00	0.00	0.00	0.00	0.00	0.00	0.00	0.00	0.00
	**Max**	79.09	14.94	18.57	12.72	39.63	44.02	39.63	82.45	0.87	5.37	39.63	2.51	82.45	82.45	0.87	70.01	82.45	10.64	35.46	82.45	97.69
	**Mean**	4.23	2.53	5.58	3.48	6.40	6.79	7.44	6.29	0.17	1.45	12.96	0.74	9.90	8.95	0.18	17.59	7.30	2.74	7.72	8.60	52.20
	**SE**	2.43	1.19	2.70	0.99	1.40	1.53	1.54	1.71	0.17	1.00	5.27	0.43	2.78	1.22	0.13	2.07	3.70	1.14	2.77	1.55	16.12
	**SD**	13.97	4.45	7.13	4.77	8.28	10.60	9.25	13.43	0.39	2.24	16.66	1.14	17.14	11.52	0.34	16.56	18.14	3.59	12.05	14.48	42.65
	**Med**	0.07	0.27	1.85	0.47	3.61	2.14	4.03	0.43	0.00	0.87	2.39	0.00	1.39	5.63	0.00	13.43	0.00	1.36	0.38	2.24	59.37
**EAG (%)**	**Min**	0.22	0.00	6.07	0.00	0.61	0.22	0.61	0.00	0.61	1.49	1.36	0.22	0.22	0.00	0.22	0.00	0.00	0.61	0.22	0.00	0.00
	**Max**	35.42	30.24	46.06	19.27	35.42	38.61	40.83	46.06	6.59	16.61	38.61	33.99	38.61	46.10	33.99	46.10	35.42	23.75	46.06	46.06	33.83
	**Mean**	14.27	8.31	23.30	7.60	9.44	12.26	14.40	14.05	2.93	7.06	18.97	18.56	15.92	16.51	14.14	20.28	14.13	10.72	18.62	14.13	12.30
	**SE**	1.77	2.30	5.88	1.17	1.31	1.44	2.02	1.40	1.07	2.75	4.01	4.90	1.77	1.09	5.82	1.43	2.19	2.09	3.20	1.22	4.96
	**SD**	10.17	8.60	15.56	5.63	7.76	9.97	12.13	11.00	2.40	6.15	12.68	12.97	10.92	10.27	15.40	11.45	10.71	6.62	13.96	11.36	13.12
	**Med**	10.45	6.94	24.68	7.28	8.68	8.57	9.63	10.07	1.95	6.59	20.10	20.93	14.78	13.48	6.59	20.22	12.19	11.85	20.93	10.20	10.47
**URB (%)**	**Min**	0.00	0.00	0.00	0.00	0.00	0.00	0.00	0.00	0.00	0.00	0.00	0.00	0.00	0.00	0.00	0.00	0.00	0.00	0.00	0.00	0.00
	**Max**	8.14	2.60	0.95	3.97	9.87	11.23	9.87	9.87	1.54	0.17	9.46	1.30	6.80	9.87	1.30	25.55	9.87	3.82	9.46	11.23	1.15
	**Mean**	0.89	0.69	0.14	0.88	1.84	2.03	1.93	0.90	0.34	0.03	2.43	0.40	1.29	1.44	0.37	2.26	0.82	0.64	0.83	1.63	0.59
	**SE**	0.30	0.22	0.13	0.28	0.38	0.38	0.36	0.22	0.30	0.03	1.12	0.22	0.29	0.21	0.22	0.59	0.42	0.37	0.50	0.25	0.17
	**SD**	1.74	0.84	0.36	1.36	2.24	2.62	2.14	1.76	0.67	0.08	3.55	0.57	1.78	2.01	0.59	4.76	2.05	1.17	2.20	2.35	0.46
	**Med**	0.05	0.19	0.00	0.05	1.10	1.27	1.30	0.17	0.00	0.00	0.35	0.05	0.91	0.95	0.00	0.75	0.00	0.18	0.00	0.70	0.70

## Appendix D

**Table A5:** Braun–Blanquet and van der Maarel cover and abundance scales.

Braun–Blanquet Code	Cover/Abundance	van der Maarel Code
r	one individual, coverage < 5%	1
+	up to 5 individuals, coverage < 5%	2
1	up to 50 individuals, coverage < 5%	3
2m	over 50 individuals, coverage < 5%	4
2a	coverage 5–15%	5
2b	coverage 15–25%	6
3	coverage 25–50%	7
4	coverage 50–75%	8
5	coverage over 75%	9

## Appendix E

**Table A6:** Descriptive statistics for all environmental variables calculated for the Pannonian and Dinaric Ecoregion. For abbreviations of environmental variables, see Table 1.

	Pannonian Ecoregion						Dinaric Ecoregion					
	Min	Max	Mean	SE	SD	Med	Min	Max	Mean	SE	SD	Med
T (°C)	7.00	25.02	13.35	0.34	2.58	12.93	7.85	18.75	12.58	0.22	2.50	12.33
pH	7.03	8.29	7.88	0.04	0.28	7.93	7.13	8.38	7.94	0.02	0.23	7.95
EC (μS/cm)	80.34	923.50	468.27	23.64	180.03	472.50	147.04	1073.00	444.81	14.82	164.39	405.20
TSS (mg/L)	1.00	49.80	13.30	1.38	10.49	10.83	0.85	45.50	3.74	0.56	6.22	1.94
DO (mgO₂/L)	4.84	14.25	9.50	0.25	1.91	9.56	7.51	12.55	10.64	0.08	0.94	10.80
ALK (mgCaCO₃/L)	48.00	446.67	235.13	11.55	87.94	246.11	77.03	344.62	206.29	4.15	46.25	200.41
BOD (mgO₂/L)	0.85	9.78	2.81	0.20	1.52	2.66	0.14	2.98	1.04	0.05	0.58	1.05
COD (mgO₂/L)	1.75	13.11	4.89	0.30	2.26	4.53	0.25	10.68	1.48	0.11	1.17	1.26
NH₄^+^ (mgN/L)	0.004	6.056	0.286	0.110	0.839	0.081	0.001	0.862	0.042	0.009	0.105	0.013
NO_2_^−^ (mgN/L)	0.001	0.296	0.025	0.005	0.042	0.011	0.000	0.215	0.010	0.002	0.023	0.003
NO_3_^−^ (mgN/L)	0.075	4.442	0.907	0.119	0.904	0.667	0.060	1.594	0.558	0.029	0.320	0.505
N_tot_ (mgN/L)	0.389	8.900	1.650	0.186	1.417	1.324	0.125	2.638	0.784	0.040	0.448	0.692
PO_4_^3−^ (mgP/L)	0.005	0.473	0.052	0.009	0.069	0.034	0.001	0.179	0.009	0.002	0.018	0.004
P_tot_ (mgP/L)	0.014	0.699	0.115	0.013	0.103	0.085	0.001	0.271	0.027	0.003	0.035	0.018
NAT (%)	0.00	100.00	53.81	3.29	25.04	53.31	0.00	99.78	76.80	1.46	16.27	80.59
IAG (%)	0.00	97.69	25.86	3.03	23.05	20.61	0.00	82.45	7.51	1.03	11.51	4.54
EAG (%)	0.00	46.06	18.54	1.63	12.38	20.01	0.00	46.10	14.03	0.86	9.58	12.22
URA (%)	0.00	25.55	1.79	0.63	4.78	0.26	0.00	11.23	1.66	0.21	2.29	0.96

**Table A7:** Descriptive statistics for all environmental variables calculated for the Continental and Mediterranean Subecoregion of the Dinaric Ecoregion. For environmental variables’ abbreviations, see Table 1.

	Continental Subecoregion						Mediterranean Ecoregion					
	Min	Max	Mean	SE	SD	Med	Min	Max	Mean	SE	SD	Med
T (°C)	7.85	17.85	12.75	0.29	2.30	12.66	8.42	18.75	12.39	0.35	2.70	12.12
pH	7.13	8.26	7.89	0.03	0.23	7.91	7.48	8.38	7.99	0.03	0.22	8.03
EC (μS/cm)	189.33	1073.00	491.21	25.52	202.58	415.80	147.04	641.75	396.08	11.57	89.65	391.57
TSS (mg/L)	0.85	45.50	4.25	0.97	7.73	2.03	1.00	23.00	3.21	0.52	4.05	1.70
DO (mgO₂/L)	7.51	12.14	10.52	0.12	1.00	10.65	8.86	12.55	10.77	0.11	0.86	10.87
ALK (mgCaCO₃/L)	90.33	344.62	205.77	6.36	50.90	197.25	77.03	281.67	206.86	5.31	41.13	205.08
BOD (mgO₂/L)	0.14	2.64	0.93	0.07	0.59	0.80	0.25	2.98	1.15	0.07	0.55	1.09
COD (mgO₂/L)	0.25	10.68	1.49	0.18	1.47	1.17	0.25	3.03	1.46	0.10	0.75	1.40
NH₄^+^ (mgN/L)	0.002	0.862	0.043	0.017	0.134	0.013	0.001	0.359	0.042	0.008	0.063	0.010
NO_2_^−^ (mgN/L)	0.000	0.215	0.012	0.004	0.030	0.003	0.001	0.061	0.007	0.002	0.012	0.003
NO_3_^−^ (mgN/L)	0.060	1.327	0.407	0.033	0.264	0.348	0.092	1.594	0.719	0.038	0.296	0.685
N_tot_ (mgN/L)	0.125	2.243	0.602	0.046	0.370	0.503	0.158	2.638	0.978	0.057	0.445	0.868
PO_4_^3−^ (mgP/L)	0.001	0.179	0.010	0.003	0.024	0.003	0.002	0.070	0.009	0.001	0.010	0.006
P_tot_ (mgP/L)	0.001	0.271	0.026	0.005	0.039	0.014	0.005	0.223	0.028	0.004	0.030	0.022
NAT (%)	21.34	99.19	76.73	2.02	16.14	80.19	0.00	99.78	76.88	2.13	16.54	81.08
IAG (%)	0.00	48.97	8.26	1.43	11.41	4.88	0.00	82.45	6.71	1.50	11.66	3.22
EAG (%)	0.61	46.10	13.04	1.18	9.45	10.92	0.00	40.83	15.09	1.25	9.68	13.58
URA (%)	0.00	11.23	1.97	0.33	2.65	0.92	0.00	9.87	1.32	0.23	1.80	0.97
